# A Case of Periaortic Lymphoma Mimicking Complicated Type B Acute Aortic Dissection: A Pitfall in the Endovascular Surgery Era

**DOI:** 10.3400/avd.cr.20-00019

**Published:** 2020-09-25

**Authors:** Yuki Imamura, Norihiro Kondo, Yoshiaki Saito, Kaoru Ogawa, Mari Chiyoya, Ikuo Fukuda

**Affiliations:** 1 Department of Thoracic and Cardiovascular Surgery, Hirosaki University Graduate School of Medicine; 2 Department of Anatomic Pathology, Hirosaki University Graduate School of Medicine

**Keywords:** thoracic endovascular aortic repair, lymphoma, unusual postoperative course

## Abstract

We report a case of periaortic lymphoma mimicking Stanford type B acute aortic dissection treated for impending rupture by thoracic endovascular aortic repair. Although no endoleak was detected, the aneurysm enlarged continuously. Repeat computed tomography scans showed that an aortic aneurysm-like structure around the stent graft had enlarged irregularly. Histopathological examination revealed diffuse large B-cell malignant lymphoma. Post-chemotherapy, the aneurysm-like structure disappeared without any fistula or rupture. In open surgery, differentiating between aneurysms and malignancy is easy under direct vision; however, in the endovascular surgery era, this is a pitfall because no surgical specimen of the lesion can be obtained.

## Introduction

Thoracic endovascular aortic repair (TEVAR) is an effective treatment for complicated Stanford type B acute aortic dissection (AAD). A symptomatic or rapidly progressive type B AAD is an absolute indication for surgical intervention. However, differentiating periaortic malignant lymphoma from a rapidly growing type B AAD^1)^ is difficult. We hereby present a case of a patient, who was subsequently diagnosed with periaortic malignant lymphoma by lymph-node biopsy, treated with TEVAR for Stanford type B AAD.

## Case Report

**Figure 1:**
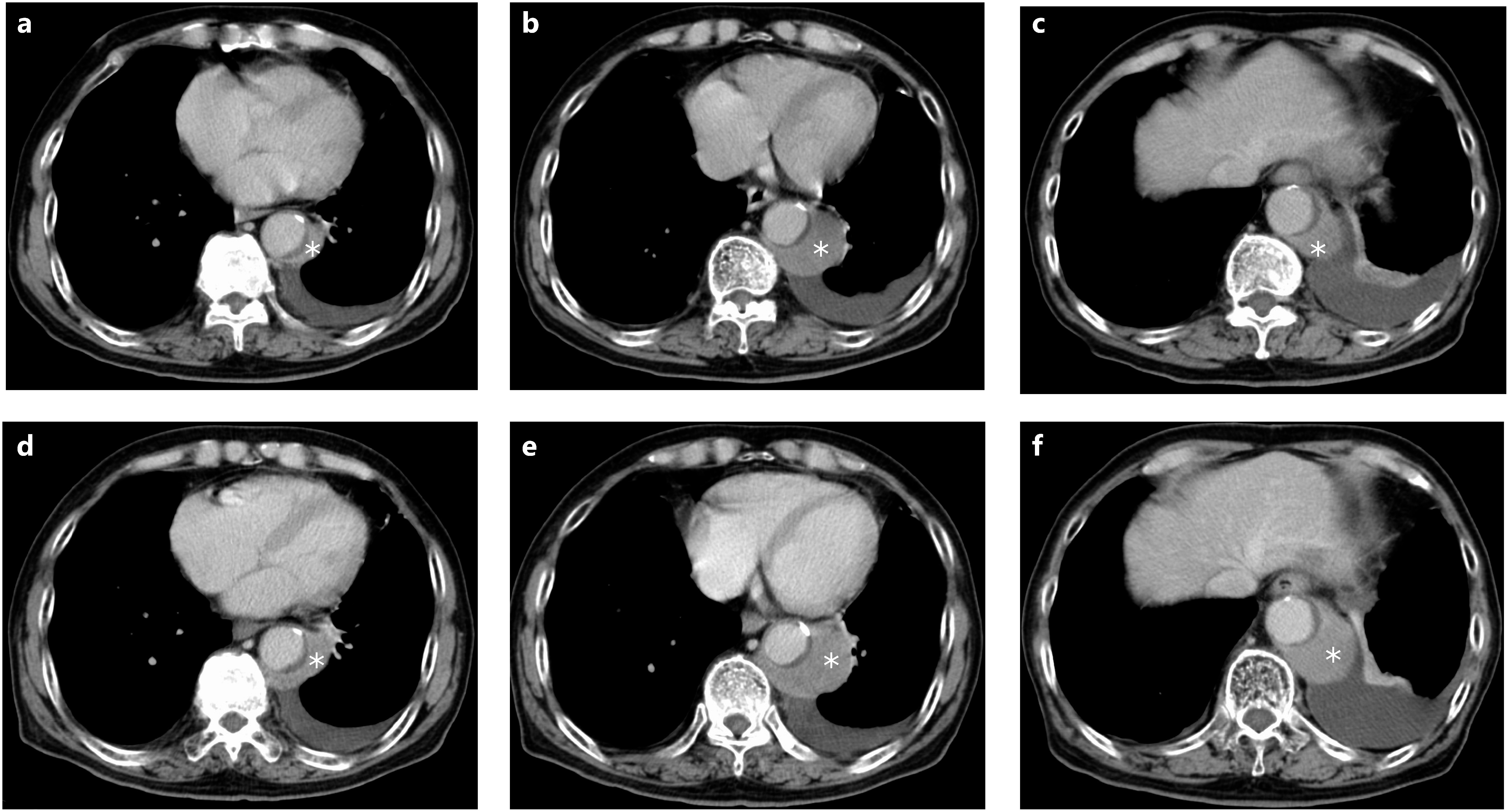
Fig. 1 Computed tomography (CT) images **a–c.** CE-CT on admission showed a false lumen-like structure. **d–f.** CE-CT showed rapid increase in the maximum diameter of the dissected aorta one month after onset. The asterisk (*) indicates a false lumen-like structure.

An 85-year-old man, who had been hospitalized with persistent low-grade back pain, was referred to our hospital after being diagnosed with Stanford type B AAD. Contrast-enhanced computed tomography (CT) scans demonstrated a false lumen-like structure and left pleural effusion, leading to the suspicion of a Stanford type B AAD (**Figs. 1a**–**1c**). The patient was medically managed with antihypertensive drugs. Although blood pressure control was performed intensively, his back pain gradually worsened. Repeated contrast-enhanced CT scans showed rapid progression of the maximum diameter of the descending aorta from 51 to 57 mm and pleural effusion increasing within one month after the onset (**Figs. 1d**–**1f**). Laboratory data were as follows: white blood cell count, 4.6×10^3^/mL; hemoglobin, 14.2 g/dL; platelet count, 120×10^3^/µL; and C-reactive protein level, 0.6 mg/dL. The level of fibrin/fibrinogen degradation products was elevated to 24.0 µg/mL.

CE-CT: contrast-enhanced computed tomography.

**Figure 2:**
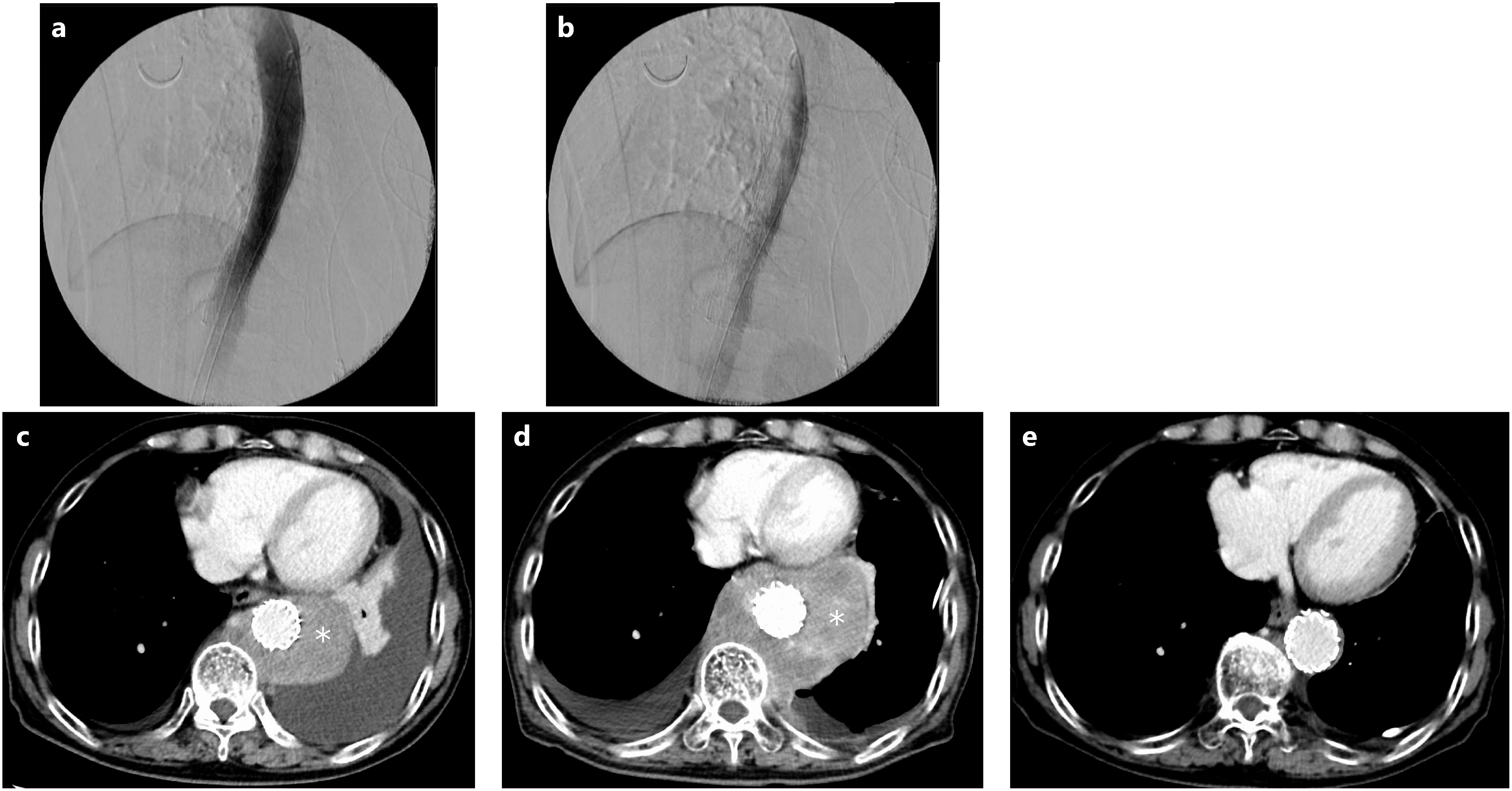
Fig. 2 Intraoperative angiography and computed tomography (CT) images. **a** and **b.** Images of the completion angiography; no endoleak nor feeding artery-like finding to the neoplasm at the early phase (**a**) and late phase (**b**). **c.** Postoperative CT showed progression of the thoracic aneurysm with increased left pleural effusion (arrowhead) without an endoleak. **d.** Re-enhanced CT showed that the aortic aneurysm around the stent graft enlarged irregularly with heterogenous contrast enhancement. **e.** The aneurysm-like structure around the stent graft disappeared without any findings of a fistula or rupture on the contrast-enhanced-CT after chemotherapy. The asterisk indicates a false lumen-like structure.

**Figure 3:**
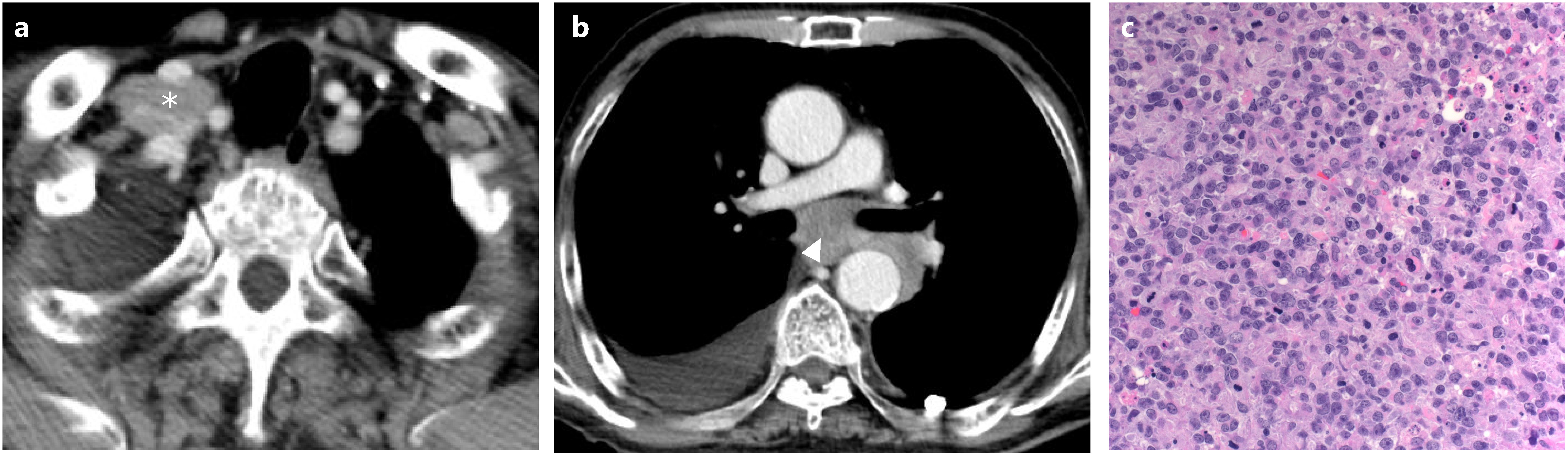
Fig. 3 Computed tomography (CT) and histopathology of the lymph nodes. **a** and **b.** Delayed enhanced CT three months after onset showing right supraclavicular lymph-node (asterisk) swelling (**a**) and bronchial bifurcation lymph-node (arrowhead) swelling (**b**). **c.** Histopathology of the biopsy specimen revealed diffuse and dense proliferation of atypical cells with large rounded or polygonal nuclei.

Since impending rupture of the aorta by dissection was considered in this case, the patient was transferred to our hospital and then underwent emergent TEVAR. The right femoral artery was exposed as an access route. A GORE Conformable TAG (W. L. Gore & Associated, Inc., Flagstaff, AZ, USA) was inserted through the DrySeal Introducer Sheath (W. L. Gore & Associated, Inc., Flagstaff, AZ, USA) into the descending thoracic aorta. The stent graft was deployed from the Th8 to Th12 levels, thereby sufficiently covering the “dissected” segment of the aorta. Intraoperative angiography revealed no endoleak (**Figs. 2a** and **2b**). He had no back pain after the operation and was discharged on postoperative day 7. One month after the operation, follow-up CT scans were performed and showed thoracic aneurysm progression from 57 to 63 mm with increase in the left pleural effusion despite no apparent endoleak (**Fig. 2c**). Since the patient complained of dyspnea, left pleural drainage was performed, which was found to be a serous effusion with an elevated leucocyte count. One month later, thoracentesis was performed again for the recurrent pleural effusion, demonstrating leucocyte and adenosine deaminase elevation in the fluid; therefore, tuberculous pleuritis was suspected. Enhanced CT of the chest was performed, demonstrating enlargement of the periaortic lesion with heterogenous contrast enhancement around the stent graft (**Fig. 2d**) and swelling of the right supraclavicular and bronchial bifurcation lymph nodes (**Figs. 3a** and **3b**). Since the plasma level of interleukin-2 was as high as 4,000 pg/mL, a malignant lymphoma was suspected and a surgical biopsy of the right supraclavicular lymph node was performed. Histopathological examination revealed diffuse proliferation of highly atypical cells with large rounded or polygonal nuclei and scant cytoplasm. Prominent nucleoli were conspicuous in many atypical cells, which were positive for CD20. Therefore, a definitive diagnosis of diffuse large B-cell lymphoma (DLBCL) was made (**Fig. 3c**). As per the protocol for non-Hodgkin’s lymphoma (R-mini CHOP protocol), the patient received chemotherapy. Then, the periaortic lesion around the stent graft had completely disappeared (**Fig. 2e**). Two and a half years after the surgery, the patient had no aortic expansion or recurrence.

The authors have obtained written informed consent from the patient for reporting this case.

## Discussion

We report a case of a TEVAR-treated patient for Stanford type B AAD, who was subsequently diagnosed with periaortic malignant lymphoma by lymph-node biopsy. Periaortic malignant lymphoma sometimes presents similar to an aortic dissection, aneurysms, and rupture.^1–3)^ Unlike open surgery, an endovascular approach does allow for a biopsy specimen of the aortic wall to be taken for histopathological examination.^4)^ Imaging examinations play a significant role in distinguishing aortic dissection from malignant lymphoma. The typical radiological features of a malignant lymphoma on CT include pleural effusions, significant thoracic lymphadenopathy, and a soft tissue mass with post-contrast enhancement in the venous phase.^5)^ In the present case, a false lumen-like structure, observed on preoperative CT, was suspected to be a rapidly growing chronic type B AAD but was later found to be a periaortic malignant lymphoma. We retrospectively reviewed the preoperative CT scans and found no intimal tear in the descending aorta as an entry or reentry. Using only CT images, neoplastic diseases are difficult to distinguish from localized aortic dissection due to their similarities in clinical symptoms and the presence of pleural effusions on CT images in both conditions.

There are several reports describing the diagnosis of aortic diseases due to invasion by periaortic malignant lymphoma after treatment by endovascular repair. Yiu et al.^6)^ reported a case in which a positron emission tomography-CT (PET-CT) scan was performed because of persistent backache despite complete endovascular treatment. Raupach et al.^7)^ reported a case in which the follow-up CT showed progressive enlargement of a circumferential periaortic hypodense mass, which had been investigated after the patient presented with fatigue and loss of weight. In the former reports, periaortic malignant lymphoma was suspected due to the unusual clinical symptoms. In the present study, back pain, which was caused by a rapid aortic expansion with degeneration of the wall by the lymphoma, as Ting et al.^8)^ demonstrated, was absent after TEVAR, of which the reason may be the retention of the diseased aortic wall with a stent graft. However, the patient had complained of persistent fatigue, and repeat CT scans showed effusions of the pleura, expansion of the descending aorta, and swelling of the right supraclavicular and bronchial bifurcation lymph nodes. Malignant lymphoma could have been diagnosed using the repeat CT scans. The patient received chemotherapy and had no aortic expansion or recurrence for two and a half years.

When investigating for differential diagnoses, magnetic resonance imaging (MRI) scans may be useful for differentiating aortic diseases from lymphoma.^1–3)^ However, although MRI might be useful during the early diagnosis, in the present case, artifacts caused by the metallic stent made the evaluation difficult after endovascular treatment. Compared to MRI, PET-CT scans play a significant role in the early diagnosis of periaortic lymphoma^9)^ and might be considered more useful for detecting periaortic pathology in cases treated by TEVAR. Overall, an unusual postoperative course after endovascular treatment for aortic dissection may need to be investigated using these imaging techniques. In open surgery, differentiating between an aneurysm and malignancy is easy under direct vision; however, in the endovascular surgery era, this is a pitfall because no surgical specimen of the lesion can be obtained. When the patient has corresponding symptoms, which is typical of aortic disease, but CT images do not show typical features, periaortic malignant lymphoma should be considered.

## Conclusion

In cases with unusual findings following TEVAR, neoplastic exploration should be considered to search for malignant lymphoma.
